# The chromosomal genome sequence of the staghorn coral, *Acropora cervicornis* (Lamarck, 1816) (Scleractinia: Acroporidae) and its associated microbial metagenome sequences

**DOI:** 10.12688/wellcomeopenres.27564.1

**Published:** 2026-09-09

**Authors:** Kara Rising, Richard Karp, Andrew Baker, Sebastian Metz, Michael Sweet, Graeme Oatley, Elizabeth Sinclair, Eerik Aunin, Noah Gettle, Camilla Santos, Michael Paulini, Haoyu Niu, Victoria McKenna, Rebecca O’Brien, Wellcome Sanger Institute Tree of Life Management, Samples and Laboratory team, Wellcome Sanger Institute Scientific Operations: Sequencing Operations, Wellcome Sanger Institute Tree of Life Core Informatics team, EBI Aquatic Symbiosis Genomics Data Portal Team, Aquatic Symbiosis Genomics Project Leadership

**Affiliations:** 1 Aquatic Research Facility, Nature-based Solutions Research Centre, University of Derby, Derby, England, UK; 2 Department of Marine Biology and Ecology, University of Miami, Coral Gables, Florida, USA; 3 Tree of Life Programme, Wellcome Sanger Institute, Hinxton, England, UK

**Keywords:** Acropora cervicornis, staghorn coral, genome sequence, chromosomal, Scleractinia, microbial metagenome assembly

## Abstract

We present a genome assembly from a specimen of *Acropora cervicornis* (staghorn coral; Cnidaria; Anthozoa; Scleractinia; Acroporidae). The genome sequence has a total length of 328.68 megabases. Most of the assembly (99.07%) is scaffolded into 14 chromosomal pseudomolecules. The mitochondrial genome has also been assembled, with a length of 18.26 kilobases. Gene annotation of this assembly by Ensembl identified 24,579 protein-coding genes. From the metagenome data, we recovered two bins, of which one was a high-quality MAG.

## Species taxonomy

Eukaryota; Opisthokonta; Metazoa; Eumetazoa; Cnidaria; Anthozoa; Hexacorallia; Scleractinia; Refertina; Acroporidae; *Acropora*; *Acropora cervicornis* (Lamarck, 1816) (NCBI:txid6130).

## Background

*Acropora cervicornis* (Lamarck, 1816) ( Figure 1), also known as the Caribbean staghorn coral, is a branching scleractinian distributed across the Caribbean Sea, Gulf of Mexico, and tropical western Atlantic, from Florida and the Bahamas to Venezuela and Colombia. It is one of three Caribbean acroporids and, together with *A. palmata* (elkhorn coral), forms the hybrid *A. prolifera* ( Van Oppen *et al.*, 2000). Colonies are characterised by antler-like branches (typically >10 cm long and 1–2 cm wide) ( Wallace *et al.*, 2012). Axial corallites are conspicuously larger and paler than radial corallites, while overall colony colour ranges from yellow to brown depending on the density of Symbiodiniaceae symbionts ( Wallace *et al.*, 2012). Linear extension can reach ~7 cm yr ^−1^, depending on geographic area and environmental conditions, making *A. cervicornis* one of the fastest-growing Caribbean corals ( Gladfelter *et al.*, 1978). A recent whole-genome assembly and annotation clarified its divergence from Indo-Pacific *Acropora*, corresponding to the closure of the Tethys Sea ( Selwyn & Vollmer, 2023). *A. cervicornis* typically hosts *Symbiodinium fitti*, but in some nearshore habitats has also been found to host *Durusdinium* ( Baums *et al.*, 2010; Thornhill *et al.*, 2006). It is a hermaphroditic broadcast spawner that typically spawns two to ten days after the full moon in July or August ( Laforest, 2023; Vargas-Ángel *et al.*, 2006).

**Figure 1.**
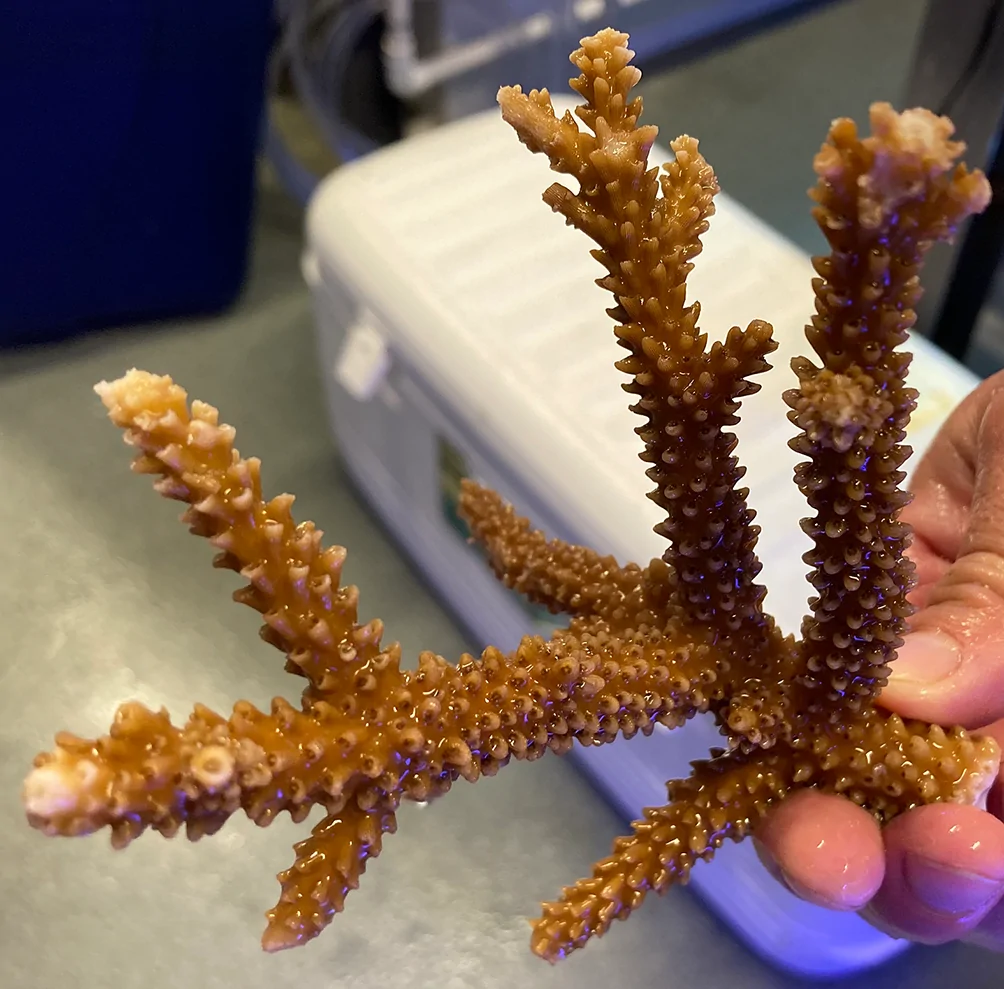
*A. cervicornis* specimen prior to sampling.

*A. cervicornis* is a foundational reef-builder with limited functional redundancy in the Caribbean and enhances shoreline protection and habitat complexity, supporting high biodiversity ( Agudo-Adriani *et al.*, 2016; Guendulain-Garcia *et al.*, 2024). However, despite its vital importance for coastal ecosystems, its populations have dwindled over the past decades due to its sensitivity, especially to hurricanes and elevated sea temperatures that cause bleaching and disease, further exacerbated by other anthropogenic stressors ( Mercado-Molina *et al.*, 2015). It is at risk of extinction due to these stressors ( Carpenter *et al.*, 2008) and has been listed as Critically Endangered on the IUCN Red List ( Aronson *et al.*, 2008). Since the 1970s, populations of *A. cervicornis* have decreased by up to 98% in some locations ( Manzello *et al.*, 2025; Miller *et al.*, 2002; Nylander-Asplin *et al.*, 2021). Efforts to restore *A. cervicornis* on reefs across the Caribbean have substantially increased over the past decades ( Mercado-Molina *et al.*, 2015), with Caribbean restoration programmes targeting it, along with *A. palmata*, in asexual and sexual propagation activities more often than their boulder-like relatives ( Boström-Einarsson *et al.*, 2020). Despite these efforts, populations have not significantly recovered in some areas, and evidence for restoration success is limited beyond two years after outplanting ( Boisvert *et al.*, 2024). These realities highlight the need for genomic resources. Here, we present a whole-genome annotation for *A. cervicornis* to support studies of adaptation, demography, and restoration performance in the face of accelerating loss.

Other assemblies for this species are also available, including the chromosome-level assembly GCA_041430625.1, submitted by Pennsylvania State University, and the scaffold-level assemblies GCA_032359415.1 and GCA_037043185.1, submitted by Northeastern University and Pennsylvania State University, respectively.

## Methods

### Sample acquisition

The original *A. cervicornis* colony was collected in 2015 from a depth of 4 m at a mid-channel patch reef (latitude 25.6763, longitude −80.0987). The colony was subsequently propagated at a depth of 9 m in the University of Miami Offshore Coral Nursery. On 2021-03-29, a specimen (specimen ID UDUK0000163, ToLID jaAcrCerv1; Figure 1) was collected from the propagated colony using bone cutters under permit SAL-21-1794-SCRP. The specimen was transported to the University of Miami, where ten samples were taken from the colony, placed in Eppendorf tubes, immediately flash-frozen and stored at −80 °C until sequencing.

### Nucleic acid extraction

Protocols for high molecular weight (HMW) DNA extraction developed at the Wellcome Sanger Institute (WSI) Tree of Life Core Laboratory are available on protocols.io ( Howard *et al.*, 2025). The jaAcrCerv1 sample was weighed and triaged to determine the appropriate extraction protocol. Tissue was disrupted using the cryogenic bead beating protocol. HMW DNA was extracted using the Manual MagAttract v3 protocol. We used centrifuge-mediated fragmentation to produce DNA fragments in the 8–10 kb range, following the Covaris g-TUBE protocol for ultra-low input (ULI). Sheared DNA was purified by automated SPRI (solid-phase reversible immobilisation). The concentration of the sheared and purified DNA was assessed using a NanoDrop spectrophotometer and Qubit Fluorometer using the Qubit dsDNA High Sensitivity Assay kit. Fragment size distribution was evaluated by running the sample on the Femto Pulse system. For this sample, the final post-shearing DNA had a Qubit concentration of 1.43 ng/μL and an average fragment size of 8.1 kb.

RNA was extracted from tissue of the jaAcrCerv1 specimen in the Tree of Life Laboratory at the WSI using the RNA Extraction: Automated MagMax™ *mir*Vana protocol. The RNA concentration was assessed using a NanoDrop spectrophotometer and a Qubit Fluorometer using the Qubit RNA Broad-Range Assay kit. Analysis of the integrity of the RNA was done using the Agilent RNA 6000 Pico Kit and Eukaryotic Total RNA assay.

### PacBio HiFi library preparation and sequencing

Library preparation and sequencing were performed at the WSI Scientific Operations core. Prior to library preparation, the DNA was fragmented to ~10 kb. Ultra-low-input (ULI) libraries were prepared using the PacBio SMRTbell® Express Template Prep Kit 2.0 and gDNA Sample Amplification Kit. Samples were normalised to 20 ng DNA. Single-strand overhang removal, DNA damage repair, and end-repair/A-tailing were performed according to the manufacturer’s instructions, followed by adapter ligation. A 0.85× pre-PCR clean-up was carried out with Promega ProNex beads.

The DNA was evenly divided into two aliquots for dual PCR (reactions A and B), both following the manufacturer’s protocol. A 0.85× post-PCR clean-up was performed with ProNex beads. DNA concentration was measured using a Qubit Fluorometer v4.0 (Thermo Fisher Scientific) with the Qubit HS Assay Kit, and fragment size was assessed on an Agilent Femto Pulse Automated Pulsed Field CE Instrument (Agilent Technologies) using the gDNA 55 kb BAC analysis kit. PCR reactions A and B were then pooled, ensuring a total mass of ≥500 ng in 47.4 μl.

The pooled sample underwent another round of DNA damage repair, end-repair/A-tailing, and hairpin adapter ligation. A 1× clean-up was performed with ProNex beads, followed by DNA quantification using the Qubit and fragment size analysis using the Agilent Femto Pulse. Size selection was performed on the Sage Sciences PippinHT system, with target fragment size determined by Femto Pulse analysis (typically 4–9 kb). Size-selected libraries were cleaned with 1.0× ProNex beads and normalised to 2 nM before sequencing.

The sample was sequenced on a Revio instrument (Pacific Biosciences, California, USA). Prepared libraries with a molarity greater than 2 nM were normalised to 2 nM, and 15 μL was used for making complexes. For libraries with a molarity below 2 nM, the available 10 μL library volume was used without normalisation. Primers were annealed and polymerases were bound to generate circularised complexes, following the manufacturer’s instructions. Complexes were purified using 1.2× SMRTbell beads, diluted to the Revio loading concentration of 200–300 pM, and spiked with a Revio sequencing internal control. The sample was sequenced on a Revio 25 M SMRT cell. SMRT Link software (Pacific Biosciences), a web-based workflow manager, was used to configure and monitor the run and to carry out primary and secondary data analysis.

### Hi-C

***Sample preparation and crosslinking***

The Hi-C sample was prepared from 20–50 mg of frozen tissue from the jaAcrCerv1 sample using the Arima-HiC v2 kit (Arima Genomics). Following the manufacturer’s instructions, tissue was fixed and DNA crosslinked using TC buffer to a final formaldehyde concentration of 2%. The tissue was homogenised using the Diagnocine Power Masher-II. Crosslinked DNA was digested with a restriction enzyme master mix, biotinylated, and ligated. Clean-up was performed with SPRISelect beads before library preparation. DNA concentration was measured with the Qubit Fluorometer (Thermo Fisher Scientific) and Qubit HS Assay Kit. The biotinylation percentage was estimated using the Arima-HiC v2 QC beads.

***Hi-C library preparation and sequencing***

Biotinylated DNA constructs were fragmented using a Covaris E220 sonicator and size selected to 400–600 bp using SPRISelect beads. DNA was enriched with Arima-HiC v2 kit Enrichment beads. End repair, A-tailing, and adapter ligation were carried out with the NEBNext Ultra II DNA Library Prep Kit (New England Biolabs), following a modified protocol where library preparation occurs while DNA remains bound to the Enrichment beads. Library amplification was performed using KAPA HiFi HotStart mix and a custom Unique Dual Index (UDI) barcode set (Integrated DNA Technologies). Depending on sample concentration and biotinylation percentage determined at the crosslinking stage, libraries were amplified with 10–16 PCR cycles. Post-PCR clean-up was performed with SPRISelect beads. Libraries were quantified using the AccuClear Ultra High Sensitivity dsDNA Standards Assay Kit (Biotium) and a FLUOstar Omega plate reader (BMG Labtech).

Prior to sequencing, libraries were normalised to 10 ng/μL. Normalised libraries were quantified again to create equimolar and/or weighted 2.8 nM pools. Pool concentrations were checked using the Agilent 4200 TapeStation (Agilent) with High Sensitivity D500 reagents before sequencing. Sequencing was performed using paired-end 150 bp reads on the Illumina NovaSeq X.

### RNA library preparation and sequencing

Libraries were prepared using the NEBNext® Ultra II Directional RNA Library Prep Kit for Illumina (New England Biolabs), following the manufacturer’s instructions. Poly(A) mRNA was isolated from 100 ng of total RNA using oligo (dT) beads, converted to cDNA, and uniquely indexed; 14 PCR cycles were performed. Libraries were prepared with an intended average fragment size of 100–300 bp. Libraries were quantified, normalised, and pooled equimolarly to a final concentration of 2.8 nM before dilution to 150 pM for loading. Sequencing was carried out on the Illumina NovaSeq X, generating paired-end reads.

### Genome assembly

Prior to assembly of the PacBio HiFi reads, a database of *k*-mer counts ( *k* = 31) was generated from the filtered reads using FastK. GenomeScope2 ( Ranallo-Benavidez *et al.*, 2020) was used to analyse the *k*-mer frequency distributions, providing estimates of genome size, heterozygosity, and repeat content.

The HiFi reads were assembled using Hifiasm ( Cheng *et al.*, 2021) with the --primary option. Haplotypic duplications were identified and removed using purge_dups ( Guan *et al.*, 2020). The Hi-C reads ( Rao *et al.*, 2014) were mapped to the primary contigs using bwa-mem2 ( Vasimuddin *et al.*, 2019), and the contigs were scaffolded in YaHS ( Zhou *et al.*, 2023) with the --break option for handling potential misassemblies. The scaffolded assemblies were evaluated using Gfastats ( Formenti *et al.*, 2022), BUSCO ( Manni *et al.*, 2021) and MerquryFK ( Rhie *et al.*, 2020).

The mitochondrial genome was assembled using MitoHiFi ( Uliano-Silva *et al.*, 2023). The MitoHiFi reference was the mitochondrial genome of *Acropora cervicornis* (NC_083272.1).

### Assembly curation

The assembly was decontaminated using the Assembly Screen for Cobionts and Contaminants ( ASCC) pipeline. TreeVal was used to generate the flat files and maps for use in curation. Manual curation was conducted primarily in PretextView and HiGlass ( Kerpedjiev *et al.*, 2018). Scaffolds were visually inspected and corrected as described by Howe *et al*. (2021). Manual corrections included 26 breaks, 65 joins, and removal of 30 haplotypic duplications. This reduced the scaffold count by 28.6%, reduced the scaffold N50 by 30.7%, and reduced the total assembly length by 1.7%. The curation process is described at sanger-tol/curation-resources . PretextSnapshot was used to generate a Hi-C contact map of the final assembly.

### Assembly quality assessment

The MerquryFK tool ( Rhie *et al.*, 2020) was run in a Singularity container ( Kurtzer *et al.*, 2017) to evaluate *k*-mer completeness and assembly quality for the primary and alternate haplotypes using the *k*-mer database ( *k* = 31) computed prior to genome assembly. The analysis outputs included assembly QV scores and completeness statistics.

The genome was analysed using the BlobToolKit pipeline, a Nextflow implementation of the earlier Snakemake version ( Challis *et al.*, 2020). PacBio reads were aligned to the assembly using minimap2 ( Li, 2018) and processed with SAMtools ( Danecek *et al.*, 2021) to generate coverage tracks. The pipeline runs BUSCO ( Manni *et al.*, 2021) using lineages identified from the NCBI Taxonomy ( Schoch *et al.*, 2020). For the three basal BUSCO lineages, eukaryota, bacteria and archaea, BUSCO genes are aligned to the UniProt Reference Proteomes database ( Bateman *et al.*, 2023) using DIAMOND BLASTp ( Buchfink *et al.*, 2021). The genome assembly is divided into chunks based on the density of BUSCO genes from the closest taxonomic lineage, and each chunk is aligned to the UniProt Reference Proteomes database using DIAMOND BLASTx. Sequences without DIAMOND BLASTx hits are extracted using seqtk and aligned to the NT database using BLASTn ( Altschul *et al.*, 1990). The outputs are consolidated into a BlobDir for visualisation in BlobToolKit. The BlobToolKit pipeline was developed using nf-core tooling ( Ewels *et al.*, 2020) and MultiQC ( Ewels *et al.*, 2016), with containerisation through Docker ( Merkel, 2014) and Singularity ( Kurtzer *et al.*, 2017).

### Metagenome assembly

The metagenome assembly was generated using MetaMDBG ( Benoit *et al.*, 2024). The resulting bin sets of each binning algorithm were optimised and refined using MAGScoT ( Rühlemann *et al.*, 2022). PROKKA ( Seemann, 2014) was used to identify tRNAs and rRNAs in each bin, CheckM ( Parks *et al.*, 2015) (checkM_DB release 2015-01-16) was used to assess bin completeness/contamination, and GTDB-Tk ( Chaumeil *et al.*, 2022) (GTDB release 214) was used to taxonomically classify bins. Taxonomic replicate bins were identified using dRep ( Olm *et al.*, 2017) with default settings (95% ANI threshold). All bins were assessed for quality and categorised as metagenome-assembled genomes (MAGs) if they met the following criteria: contamination ≤5%, presence of 5S, 16S, and 23S rRNA genes, at least 18 unique tRNAs, and either ≥90% completeness or ≥ 50% completeness with fully circularised chromosomes ( Bowers *et al.*, 2017). Bins that did not meet these thresholds, or were identified as taxonomic replicates of MAGs, were retained as ‘binned metagenomes’ provided they had ≥50% completeness and ≤ 10% contamination.

## Genome sequence report

### Sequence data

PacBio sequencing of the *Acropora cervicornis* specimen generated 77.54 Gb (gigabases) from 9.38 million reads, which were used to assemble the genome. GenomeScope2 analysis using a *p* = 2 model gave a *k*-mer-based haploid genome size estimate of 297.29 Mb, with heterozygosity of 0.47% and repeat content of 24.80%; the full-model fit was 84.48% ( Figure 2). These estimates guided expectations for the assembly. The sequencing data provided approximately 235× coverage. Hi-C sequencing produced 180.84 Gb from 598.82 million reads, which were used to scaffold the assembly. RNA sequencing data were also generated and are available in public sequence repositories. Table 1 summarises the specimen and sequencing details.

**Figure 2.**
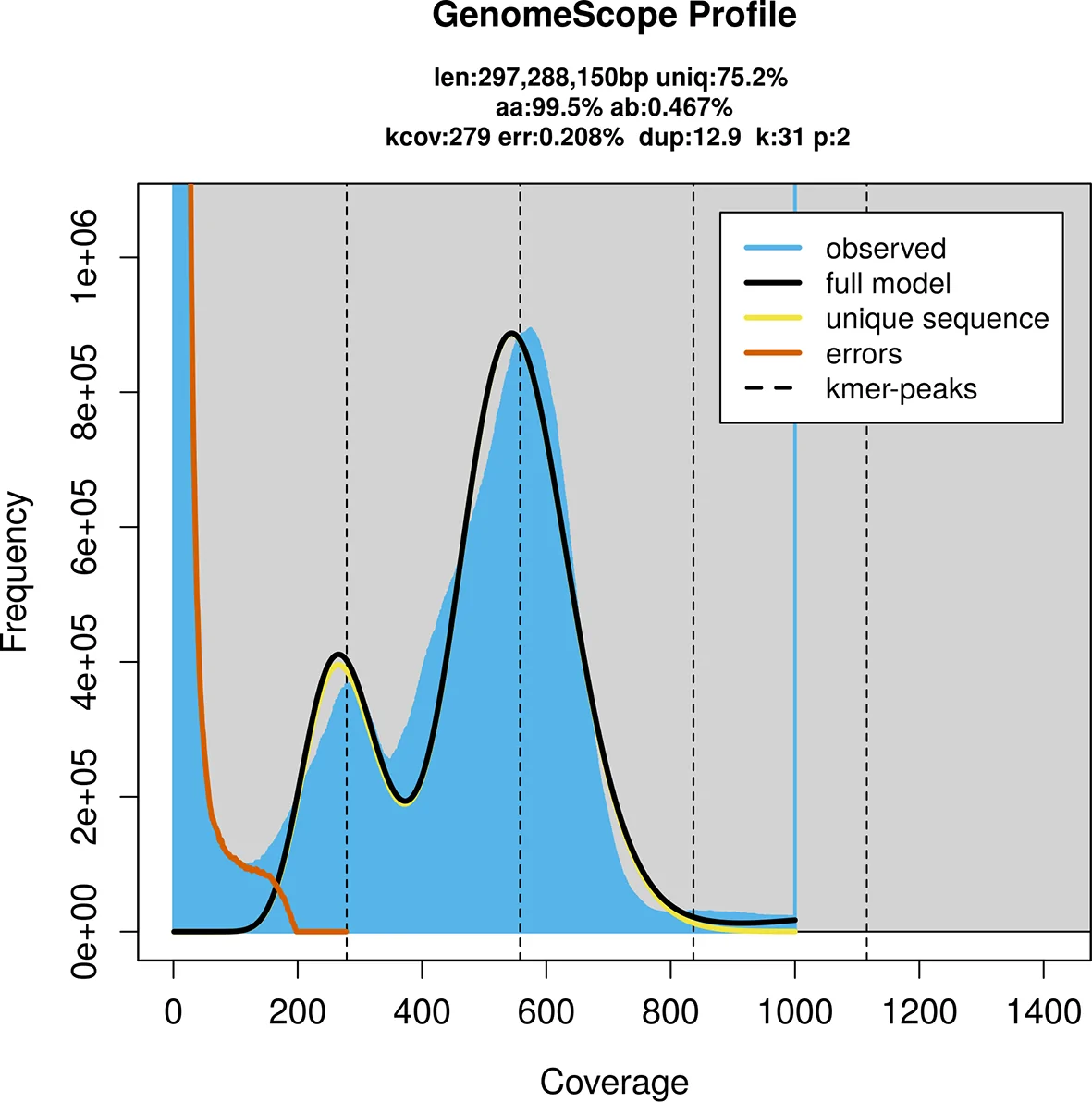
Frequency distribution of *k*-mers generated using GenomeScope2. The plot shows observed and modelled *k*-mer spectra, providing estimates of genome size, heterozygosity, and repeat content based on unassembled sequencing reads.

**Table 1.** Specimen and sequencing data for *Acropora cervicornis* (BioProject PRJEB74710).

Platform	PacBio HiFi	Hi-C	RNA-seq
**ToLID**	jaAcrCerv1	jaAcrCerv1	jaAcrCerv1
**Specimen ID**	UDUK0000163	UDUK0000163	UDUK0000163
**BioSample (source individual)**	SAMEA114784735	SAMEA114784735	SAMEA114784735
**BioSample (tissue)**	SAMEA114784749	SAMEA114784748	SAMEA114784747
**Instrument**	Revio	Illumina NovaSeq X	Illumina NovaSeq X
**Run accessions**	ERR12875177	ERR12893026	ERR13148258
**Read count total**	9.38 million reads	598.82 million read pairs	43.26 million read pairs
**Base count total**	77.54 Gb	180.84 Gb	13.06 Gb

### Assembly statistics

The primary haplotype was assembled, and contigs corresponding to an alternate haplotype were also deposited in INSDC databases. The final assembly has a total length of 328.68 Mb in 159 scaffolds, with 504 gaps, and a scaffold N50 of 22.2 Mb ( Table 2).

**Table 2.** Genome assembly data for *Acropora cervicornis.*

Genome assembly	Primary assembly
**Assembly name**	jaAcrCerv1.1
**Assembly accession**	GCA_964034985.1
**Alternate haplotype accession**	GCA_964034795.1
**Assembly level**	chromosome
**Span (Mb)**	328.68
**Number of chromosomes**	14
**Number of contigs**	663
**Contig N50 (Mb)**	1.07
**Number of scaffolds**	159
**Scaffold N50 (Mb)**	22.2
**Organelles**	Mitochondrial genome: 18.26 kb

Most of the assembly sequence (99.07%) was assigned to 14 chromosome-level scaffolds. These chromosome-level scaffolds, confirmed by Hi-C data, are named according to size ( Figure 3; Table 3).

**Figure 3.**
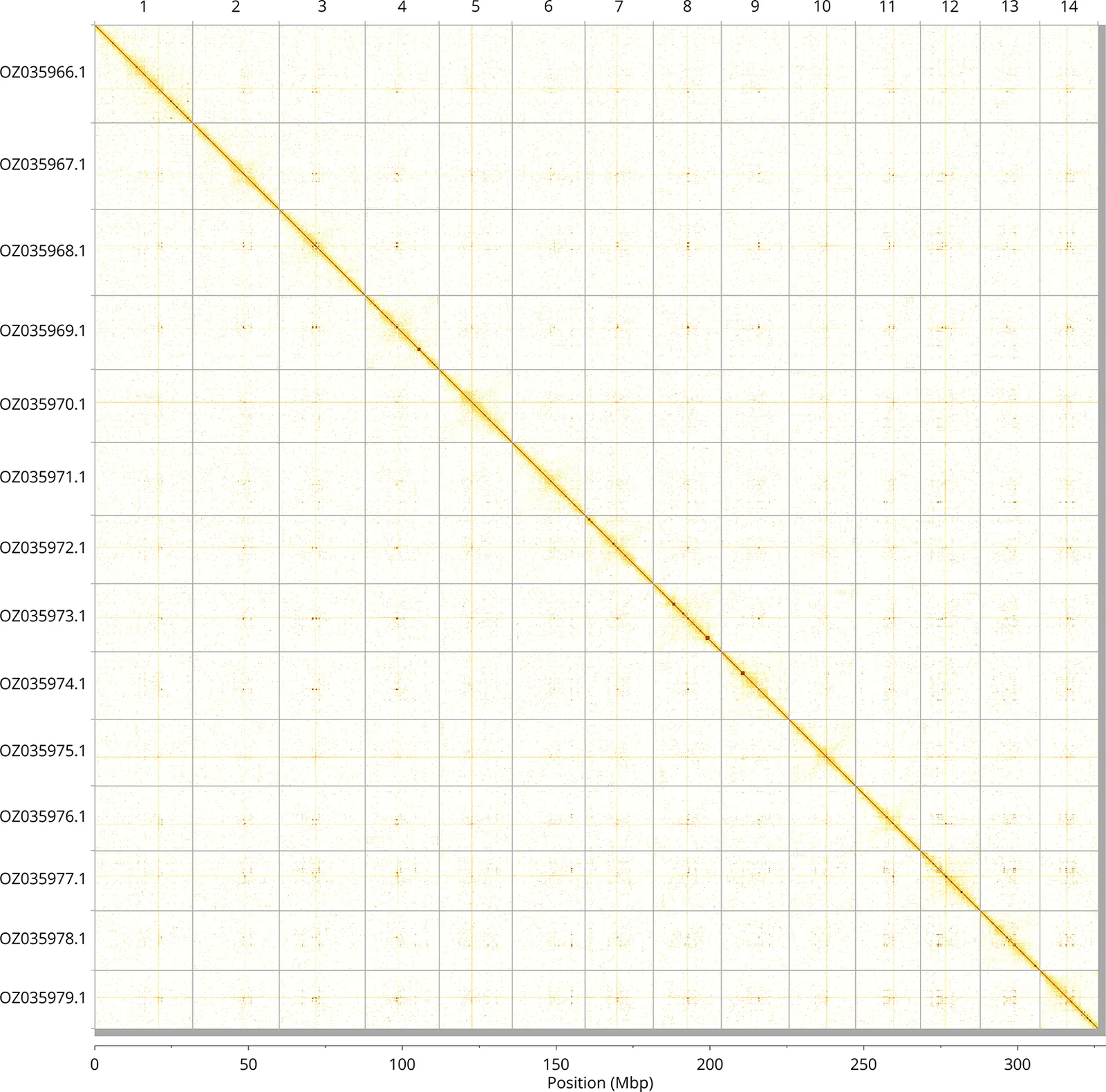
Hi-C contact map of the *Acropora cervicornis* genome assembly. Assembled chromosomes are shown in order of size and labelled along the axes, with a megabase scale shown below. The plot was generated using PretextSnapshot.

**Table 3.** Chromosomal pseudomolecules in the primary genome assembly of *Acropora cervicornis* jaAcrCerv1.

INSDC accession	Molecule	Length (Mb)	GC%
OZ035966.1	1	31.86	39
OZ035967.1	2	28.04	39
OZ035968.1	3	27.92	39
OZ035969.1	4	23.98	39
OZ035970.1	5	23.70	39
OZ035971.1	6	23.59	39
OZ035972.1	7	22.20	39
OZ035973.1	8	22.07	39
OZ035974.1	9	21.94	39
OZ035975.1	10	21.54	39
OZ035976.1	11	21.06	39
OZ035977.1	12	19.40	39
OZ035978.1	13	19.38	39
OZ035979.1	14	18.96	39

The mitochondrial genome was also assembled (length 18.26 kb, OZ035980.1). This sequence is included as a contig in the multifasta file of the genome submission and as a standalone record.

### Assembly quality metrics

The combined primary and alternate assemblies achieve an estimated QV of 56.6. The *k*-mer completeness is 90.61% for the primary assembly, 80.65% for the alternate haplotype, and 97.76% for the combined assemblies.

BUSCO v.5.5.0 analysis using the metazoa_odb10 reference set ( *n* = 954) identified 93.6% of the expected gene set (single = 93.3%, duplicated = 0.3%). The snail plot in Figure 4 summarises the scaffold length distribution and other assembly statistics for the primary assembly. The blob plot in Figure 5 shows the distribution of scaffolds by GC proportion and coverage.

**Figure 4.**
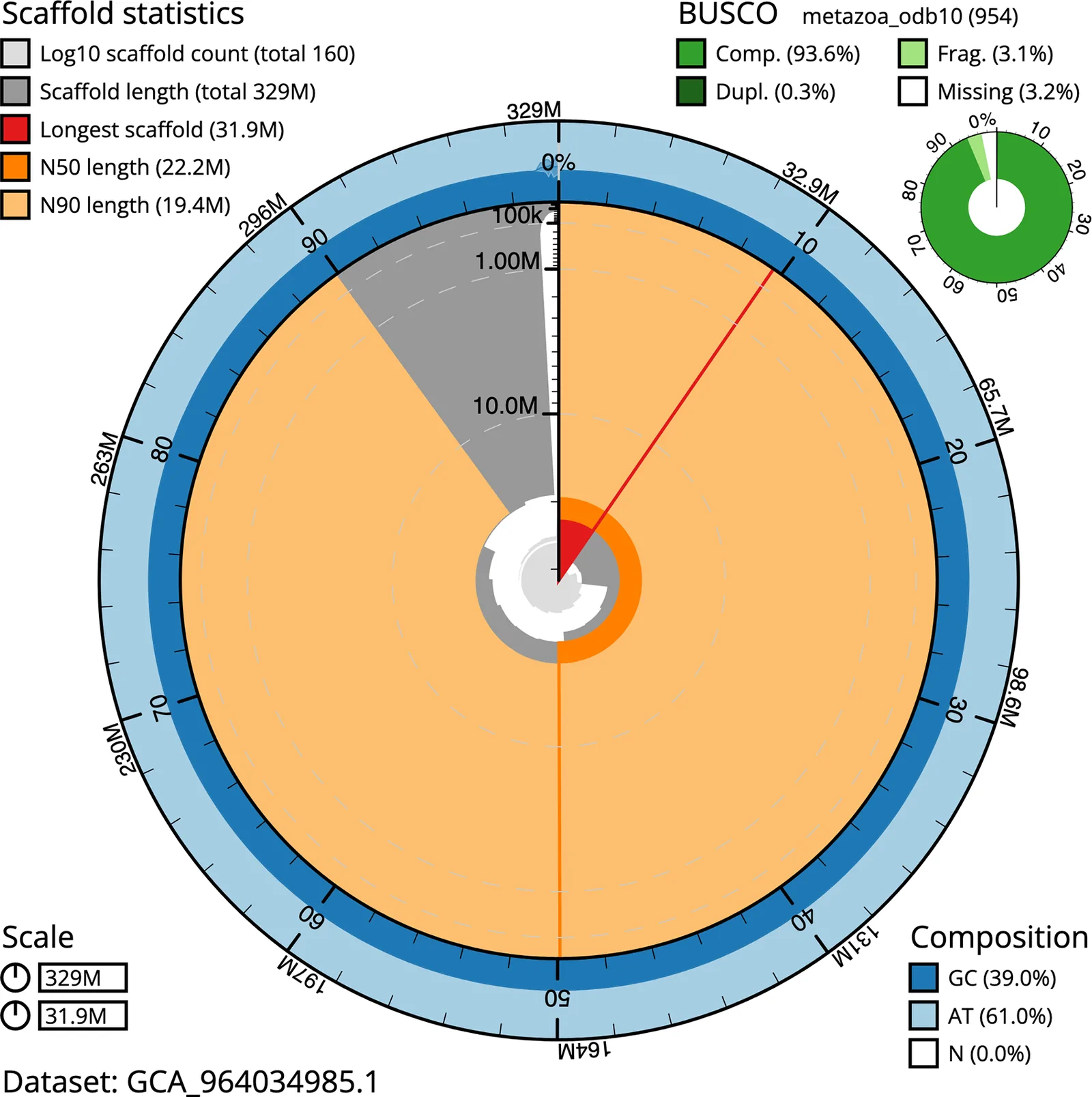
Assembly metrics for jaAcrCerv1.1. The BlobToolKit snail plot provides an overview of assembly metrics and BUSCO gene completeness. The circumference represents the length of the whole genome sequence, and the main plot is divided into 1 000 bins around the circumference. The outermost blue tracks display the distribution of GC, AT, and N percentages across the bins. Scaffolds are arranged clockwise from longest to shortest and are depicted in dark grey. The longest scaffold is indicated by the red arc, and the deeper orange and pale orange arcs represent the N50 and N90 lengths. A light grey spiral at the centre shows the cumulative scaffold count on a logarithmic scale. A summary of complete, fragmented, duplicated, and missing BUSCO genes in the metazoa_odb10 set is presented at the top right. An interactive version of this figure can be accessed on the BlobToolKit viewer.

**Figure 5.**
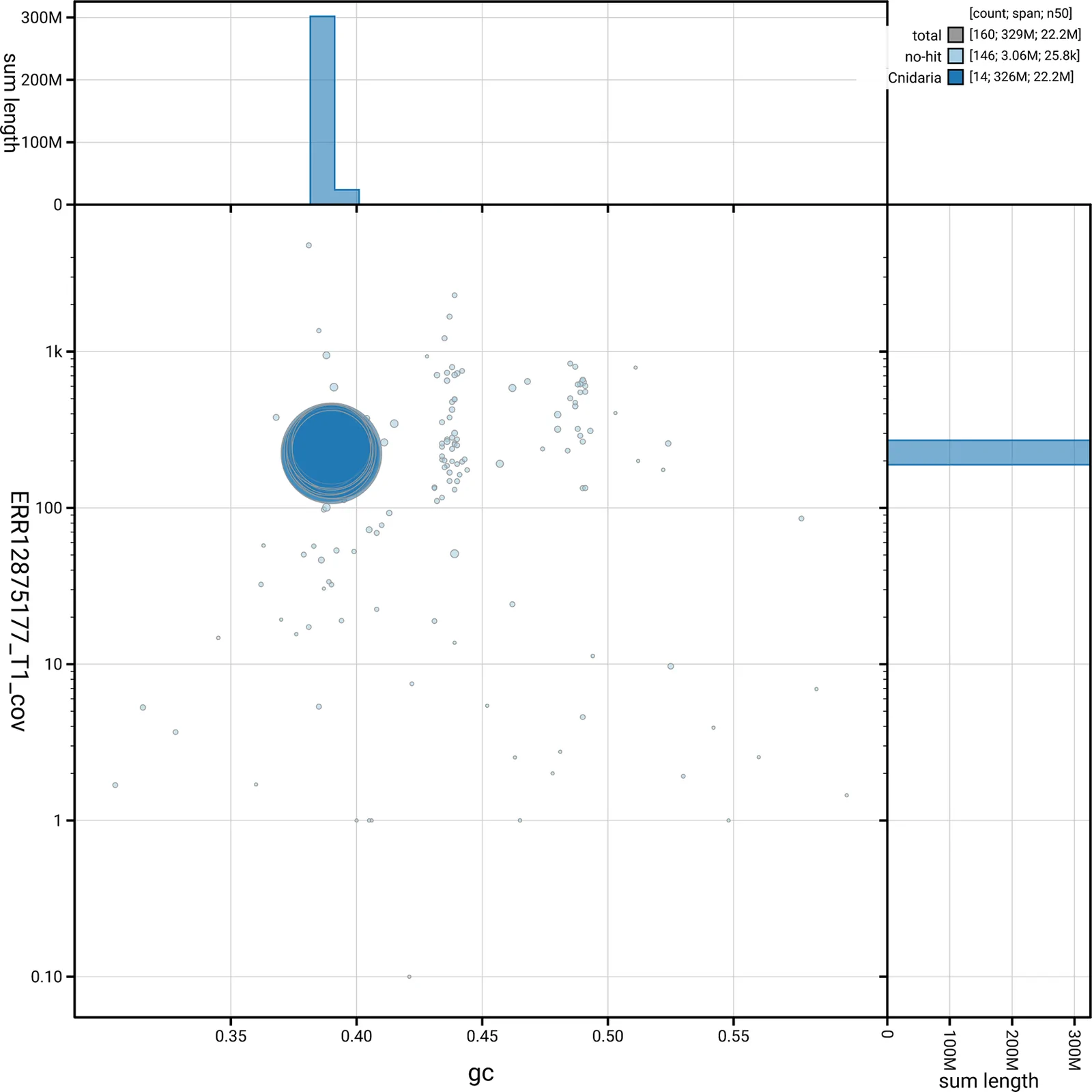
BlobToolKit blob plot for jaAcrCerv1.1. The plot shows base coverage (vertical axis) and GC content (horizontal axis). The circles represent scaffolds, with the size proportional to scaffold length and the colour representing phylum membership. The histograms along the axes display the total length of sequences distributed across different levels of coverage and GC content. An interactive version of this figure is available on the BlobToolKit viewer.

Table 4 lists the assembly metric benchmarks adapted from Rhie *et al.* (2021) and the Earth BioGenome Project (EBP) Report on Assembly Standards January 2026. The EBP metric, calculated for primary assembly, is **6.C.Q59**, meeting the recommended 6.C.Q40 reference standard.

**Table 4.** Earth BioGenome Project summary metrics for the *Acropora cervicornis* assembly.

Measure	Value	Benchmark
EBP summary (primary)	6.C.Q59	6.C.Q40
Contig N50 length	1.07 Mb	≥ 1 Mb
Scaffold N50 length	22.20 Mb	= chromosome N50
Consensus quality (QV)	Primary: 59.6; alternate: 55.1; combined: 56.6	≥ 40
*k*-mer completeness	Primary: 90.61%; alternate: 80.65%; combined: 97.76%	> 90%
BUSCO	C:93.6% [S:93.3%, D:0.3%], F:3.1%, M:3.2%, n:954	S > 90%; D < 5%
Percentage of assembly assigned to chromosomes	99.07%	≥ 90%

*Note*: The EBP summary uses log10(Contig N50); chromosome-level (C) or log10(Scaffold N50); Q (Merqury QV). BUSCO: C = complete; S = single-copy; D = duplicated; F = fragmented; M = missing; n = orthologues

### Genome annotation report

The *Acropora cervicornis* genome assembly (GCA_964034985.1) was annotated by Ensembl at the European Bioinformatics Institute (EBI). This annotation includes 59,275 transcripts from 24,579 protein-coding and 16,561 non-coding genes. The average transcript length is 6,957.82 bp, with an average of 1.44 coding transcripts per gene and 6.26 exons per transcript. Further details of this annotation are available from the Ensembl annotation page.

The *A. cervicornis* genome GCA_964034985.1 was also annotated by Metz *et al.* (2026) using BRAKER3 v3.0.8 ( Gabriel *et al.*, 2024). The annotation comprises 32,705 coding sequences, with an average gene length of 1,431 bp, 1.15 coding transcripts per gene, and 6.78 exons per transcript. Annotation completeness was assessed using BUSCO v5.8.2 against the cnidaria_odb12 lineage dataset, yielding 97.7% complete (83.2% single-copy, 14.5% duplicated), 0.8% fragmented, and 1.5% missing BUSCOs. Annotation files are available from the GitHub repository.

### Metagenome report

We recovered bins of two bacterial phyla from the metagenome assembly ( Table 5), of which one met the criteria for a MAG.

**Table 5.** Quality metrics and taxonomic assignments of the binned metagenomes.

NCBI taxon	GTDB taxonomy	Quality	Size (bp)	Contigs	Circular	Mean coverage	Completeness (%)	Contamination (%)
Spirochaetaceae bacterium	Spirochaetia	Medium	1,381,858	58	No	2.70	67.20	2
Candidatus Aquarickettsia rohweri	Alphaproteobacteria	High	1,724,599	34	No	123.64	98.90	1.10

**Table 6.** Software versions and sources used for *Acropora cervicornis.*

Software	Version	Source
BLAST	2.14.0	https://ftp.ncbi.nlm.nih.gov/blast/executables/blast+/
blobtk	0.5.1	https://github.com/genomehubs/blobtk
BlobToolKit	4.3.9	https://github.com/genomehubs/blobtoolkit
BUSCO	5.5.0; 6.1.0	https://gitlab.com/ezlab/busco
bwa-mem2	2.2.1	https://github.com/bwa-mem2/bwa-mem2
CheckM	1.1.6	https://ecogenomics.github.io/CheckM/
DIAMOND	2.1.8	https://github.com/bbuchfink/diamond
dRep	3.4.0	https://github.com/MrOlm/drep
ete3	3.1.3	https://etetoolkit.org/
fasta_windows	0.2.4	https://github.com/tolkit/fasta_windows
FastK	1.1	https://github.com/thegenemyers/FASTK
GenomeScope2.0	2.0.1	https://github.com/tbenavi1/genomescope2.0
Gfastats	1.3.6	https://github.com/vgl-hub/gfastats
GTDB-Tk	1.2.1	https://github.com/Ecogenomics/GTDBTk
Hifiasm	0.19.8-r603	https://github.com/chhylp123/hifiasm
HiGlass	1.13.4	https://github.com/higlass/higlass
MAGScoT	1.0.0	https://github.com/ikmb/MAGScoT
matplotlib	3.10.3	https://matplotlib.org/
MerquryFK	1.1.0-c1	https://github.com/thegenemyers/MERQURY.FK
MetaMDBG	Pre-release	https://github.com/GaetanBenoitDev/metaMDBG
Minimap2	2.24-r1122	https://github.com/lh3/minimap2
MitoHiFi	3.2.2	https://github.com/marcelauliano/MitoHiFi
MultiQC	1.14; 1.17 and 1.18	https://github.com/MultiQC/MultiQC
Nextflow	23.10.0; 25.10.0; 25.10.4	https://github.com/nextflow-io/nextflow
PretextSnapshot	0.0.6	https://github.com/sanger-tol/PretextSnapshot
PretextView	1.0.3	https://github.com/sanger-tol/PretextView
Prokka	1.14.5	https://github.com/tseemann/prokka
purge_dups	1.2.3	https://github.com/dfguan/purge_dups
samtools	1.16.1; 1.17; 1.18; 1.19.2; 1.6	https://github.com/samtools/samtools
sanger-tol/ascc	0.1.0	https://github.com/sanger-tol/ascc
sanger-tol/blobtoolkit	0.6.0	https://github.com/sanger-tol/blobtoolkit
sanger-tol/curationpretext	1.4.2	https://github.com/sanger-tol/curationpretext
Seqtk	1.4-r122	https://github.com/lh3/seqtk
Singularity	3.9.0	https://github.com/sylabs/singularity
TreeVal	1.1.0	https://github.com/sanger-tol/treeval
YaHS	1.1a.2	https://github.com/c-zhou/yahs

## Author information

Contributors are listed at the following links:

• Members of the Wellcome Sanger Institute Tree of Life Management, Samples and Laboratory team
• Members of Wellcome Sanger Institute Scientific Operations – Sequencing Operations
• Members of the Wellcome Sanger Institute Tree of Life Core Informatics team
• Members of the EBI Aquatic Symbiosis Genomics Data Portal Team
• The Aquatic Symbiosis Genomics Project leadership

## Wellcome Sanger Institute – Legal and Governance

The materials that have contributed to this genome note have been supplied by a Tree of Life collaborator. The Wellcome Sanger Institute employs a process whereby due diligence is carried out proportionate to the nature of the materials themselves, and the circumstances under which they have been/are to be collected and provided for use. The purpose of this is to address and mitigate any potential legal and/or ethical implications of receipt and use of the materials as part of the research project, and to ensure that in doing so we align with best practice wherever possible.

The overarching areas of consideration are:

• Ethical review of provenance and sourcing of the material
• Legality of collection, transfer and use (national and international)

Each transfer of samples is undertaken according to a Research Collaboration Agreement or Material Transfer Agreement entered into by the Tree of Life collaborator, Genome Research Limited (operating as the Wellcome Sanger Institute) and in some circumstances other Tree of Life collaborators.

## Data Availability

European Nucleotide Archive: Acropora cervicornis (staghorn coral). Accession number PRJEB74710; https://identifiers.org/ena.embl/PRJEB74710. The genome sequence is released openly for reuse. The *Acropora cervicornis* genome sequencing initiative is part of the Aquatic Symbiosis Genomics Project ( PRJEB43743) and the Sanger Institute Tree of Life Programme ( PRJEB43745). All raw sequence data and the assembly have been deposited in INSDC databases. Raw data and assembly accession identifiers are reported in Tables 1 and 2. Production code used in genome assembly at the WSI Tree of Life is available at https://github.com/sanger-tol . Table 6 lists software versions used in this study.
