# Supplementary material for: The chromosomal genome sequence of the staghorn coral, Acropora cervicornis (Lamarck, 1816) (Scleractinia: Acroporidae) and its associated microbial metagenome sequences
Source: Wellcome Open Res. 2026 Sep 9;11:613. [Version 1] doi: 10.12688/wellcomeopenres.27564.1 (PMC13639348; doi:10.12688/wellcomeopenres.27564.1)
Supplement: Peer review history

# Reviewer report: Reviewer response for version 1

**DOI:** 10.21956/wellcomeopenres.30345.r168108

**Published:** 2026-10-08

**Authors:** Nuria P Torres-Aguila

**Affiliations:** 1 Universitat de Barcelona, Barcelona, Spain

The Data Note submitted by K. Rising et al. presents a newly assembled genome of the staghorn coral species *Acropora cervicornis (Lamarck, 1816)*. The manuscript is well structured and provides sufficient detail regarding the genome assembly.

 Nevertheless, I believe that there are a couple of potentially useful details that could further improve the manuscript. First, the authors report two different **annotations**; I believe that a comparison of the gene content between these annotations would be useful to assess the extent of their overlap and to determine whether a merged annotation would be more useful to the scientific community. Second, a comparison with the **previously published genome assemblies**, mentioned in the Background section, would help to put any improvements in the quality of the new assembly into context.

 Overall, I consider this Data Note to be of good quality and of interest to the scientific community. I therefore recommend acceptance in Wellcome Open Research, with the minor comments above to be considered by the authors.

Are sufficient details of methods and materials provided to allow replication by others?

Yes

Is the rationale for creating the dataset(s) clearly described?

Yes

Are the datasets clearly presented in a useable and accessible format?

Yes

Are the protocols appropriate and is the work technically sound?

Yes

Reviewer Expertise:

Genomics, Transcriptomics.

I confirm that I have read this submission and believe that I have an appropriate level of expertise to confirm that it is of an acceptable scientific standard, however I have significant reservations, as outlined above.

# Reviewer report: Reviewer response for version 1

**DOI:** 10.21956/wellcomeopenres.30345.r168109

**Published:** 2026-10-05

**Authors:** Jayan Duminda M Senevirathna

**Affiliations:** 1 Uva Wellassa University, Badulla, Uva Province, Sri Lanka

The authors have successfully generated a genome assembly for *Acropora cervicornis* (staghorn coral; Cnidaria; Anthozoa; Scleractinia; Acroporidae), achieving approximately 93% BUSCO completeness. The assembly has been generated at the scaffold level, with the identification of pseudochromosomal structures, representing an important contribution to the genomic resources available for this species. Furthermore, the annotation of approximately 24,579 protein-coding genes provides a substantial and valuable genomic resource for future studies of the biology, evolution, population genetics, and conservation of *A. cervicornis*.

 Overall, the genome assembly and annotation appear to be of good quality and provide a comprehensive genomic resource for the species. The study represents a valuable contribution to coral genomics, and I consider the manuscript suitable for acceptance in its current form.

Are sufficient details of methods and materials provided to allow replication by others?

Yes

Is the rationale for creating the dataset(s) clearly described?

Yes

Are the datasets clearly presented in a useable and accessible format?

Yes

Are the protocols appropriate and is the work technically sound?

Yes

Reviewer Expertise:

Aquatic genetics and biotechnology

I confirm that I have read this submission and believe that I have an appropriate level of expertise to confirm that it is of an acceptable scientific standard.

# Reviewer report: Reviewer response for version 1

**DOI:** 10.21956/wellcomeopenres.30345.r168114

**Published:** 2026-10-01

**Authors:** Yichen Dai

**Affiliations:** 1 Fudan University, Shanghai, China

The authors describe a chromosome-level genome assembly and associated microbial metagenome for the staghorn coral *Acropora cervicornis* . Overall, the genome note is valuable. I only have one minor issue:

 The BRAKER3 annotation is reported as 97.7% complete, but 14.5% duplicated BUSCOs. The assembly itself has only 0.3% duplicated BUSCOs. A 14.5% duplication rate is quite high and indicates possible contamination, misannotation, or retained haplotigs. Is it possible that BUSCO was run on all predicted isoforms, not one representative isoform per gene? Do the authors have any speculation on why this happened?

Are sufficient details of methods and materials provided to allow replication by others?

Yes

Is the rationale for creating the dataset(s) clearly described?

Yes

Are the datasets clearly presented in a useable and accessible format?

Yes

Are the protocols appropriate and is the work technically sound?

Yes

Reviewer Expertise:

Genome assembly, Comparative genomics

I confirm that I have read this submission and believe that I have an appropriate level of expertise to confirm that it is of an acceptable scientific standard.

# Reviewer report: Reviewer response for version 1

**DOI:** 10.21956/wellcomeopenres.30345.r168110

**Published:** 2026-09-29

**Authors:** Alexander Klimovich

**Affiliations:** 1 Christian-Albrechts University of Kiel, Kiel, Germany

**Summary**

 This Data Note reports a chromosome-level genome assembly for the staghorn coral *Acropora cervicornis*, together with basic assembly statistics, gene annotation, and associated microbial metagenome sequences. As with comparable recent genome notes in cnidarians, it is a useful addition to coral genomic resources, supporting future work in conservation genomics, symbiosis research, and comparative genomics within Acroporidae.

**Strengths**

 The rationale for generating the resource is well articulated, and the methodology is described in great detail, enabling the reader to follow the analytical workflow. The dataset itself (genome sequence, annotation, and metagenome) can become a valuable addition to the field, provided the authors clarify its added value relative to existing resources (see point #1 below).

**Suggestions for improvement**

1. **Relationship to existing assemblies.** As the authors note in the Introduction, scaffold- and chromosome-level assemblies of *A. cervicornis* already exist. Given this, the Data Note would benefit from a more substantive comparison with these prior resources: specifically, how the new assembly compares in terms of contiguity, completeness, and annotated gene content, and what this new assembly adds beyond what is already publicly available. As presented, the added value relative to existing resources is not entirely clear to the reader.
2. **Genome features are consistent with known scleractinian biology, and this could be stated explicitly.** The reported genome size (~300 Mb), gene count (~25,000 protein-coding genes), and organization into 14 chromosomes are all in line with previously published *Acropora* genomes and karyotypes. The authors could mention this consistency explicitly, to help the reader see emerging pattern beyond individual values.
3. **Absence of the photosymbiont genome.** It is somewhat surprising that no *Symbiodinium* (or other photosymbiont) genome was recovered alongside the coral host. Please clarify whether aposymbiotic, bleached tissue was used for gDNA extraction, or whether photosymbiont reads were computationally filtered during assembly. If neither applies and *Symbiodinium* sequence is in fact present in the raw data, including it as an additional resource would add considerable value, given its role as a common holobiont member.
4. **Chromosome-size uniformity and Hi-C contact pattern.** The chromosomes appear very uniform in size (the largest, chr. 1, is not even twice the size of the smallest, chr. 14). This feature is, again, not unusual for the genus. The Hi-C contact map shows however a striking pattern: beyond the main diagonal, essentially every inter-chromosomal square shows a sharp focal dot, often with a faint "plus" shape. This suggests a substantial centromere clustering, potentially a Rabl-like configuration. I noticed that a similar pattern is evident in the genomes of other cnidarians, such as the spiny sea fan *Muricea muricata* or the Caribbean fire coral, *Millepora alcicornis*. Does this suggest that in cnidarians generally the centromeres tend to cluster in the interphase? I would encourage the authors to draw explicit attention to this observation, and, if available, to provide orthogonal evidence, such as chromosome imaging.

**Recommendation**

 I recommend the authors better position it relative to existing assemblies and address the centromere-clustering observation.

Are sufficient details of methods and materials provided to allow replication by others?

Partly

Is the rationale for creating the dataset(s) clearly described?

Yes

Are the datasets clearly presented in a useable and accessible format?

Yes

Are the protocols appropriate and is the work technically sound?

Yes

Reviewer Expertise:

Cnidarian genomics and evolutionary developmental biology; Comparative genomics; novel genes.

I confirm that I have read this submission and believe that I have an appropriate level of expertise to confirm that it is of an acceptable scientific standard, however I have significant reservations, as outlined above.
